# Dynamic Covalent Bond-Based Polymer Chains Operating Reversibly with Temperature Changes

**DOI:** 10.3390/molecules29143261

**Published:** 2024-07-10

**Authors:** Sojeong Roh, Yeonjeong Nam, My Thi Ngoc Nguyen, Jae-Hee Han, Jun Seop Lee

**Affiliations:** Department of Materials Science and Engineering, Gachon University, 1342 Seongnam-daero, Sujeong-gu, Seongnam-si 13120, Gyeonggi-do, Republic of Korea; hihithwjd@gachon.ac.kr (S.R.); nyj1717@gachon.ac.kr (Y.N.); ngocmy@gachon.ac.kr (M.T.N.N.); jhhan388@gachon.ac.kr (J.-H.H.)

**Keywords:** dynamic covalent bonds, reversibility, temperature control, equilibrium, synthesis, application, shape memory, self-healing

## Abstract

Dynamic bonds can facilitate reversible formation and dissociation of connections in response to external stimuli, endowing materials with shape memory and self-healing capabilities. Temperature is an external stimulus that can be easily controlled through heat. Dynamic covalent bonds in response to temperature can reversibly connect, exchange, and convert chains in the polymer. In this review, we introduce dynamic covalent bonds that operate without catalysts in various temperature ranges. The basic bonding mechanism and the kinetics are examined to understand dynamic covalent chemistry reversibly performed by equilibrium control. Furthermore, a recent synthesis method that implements dynamic covalent coupling based on various polymers is introduced. Dynamic covalent bonds that operate depending on temperature can be applied and expand the use of polymers, providing predictions for the development of future smart materials.

## 1. Introduction

Dynamic bonding-based polymers have recently attracted much attention as materials that can reversibly respond to changes in environmental conditions [1,2,3]. The structure and properties of polymers may change in response to an external stimulus. This has the potential to be used in various industrial fields in the future. Dynamic bonding enables structural rearrangement of polymer networks under certain conditions, typically with various combinations capable of reversible destruction and recovery at equilibrium [4,5]. For this reason, introducing dynamic and exchangeable bonds can endow polymers with self-healing, enhanced toughness, or adaptability to the material. Many laboratories have tried to provide a possible use for dynamic bonds, substituting some of the covalent bonds or crosslinking in a wide variety of polymer applications, including composites, hydrogels, and shape-memory polymers.

**Figure 1:**
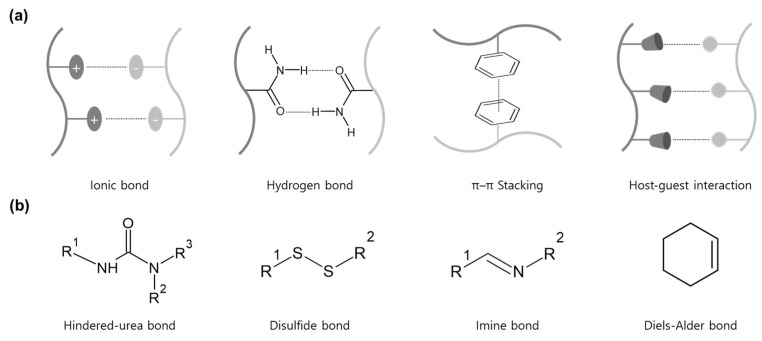
Dynamic bonding types according to bonding formation position: (**a**) Intermolecular and (**b**) Intramolecular bonds.

Dynamic bonds are divided into intermolecular and intramolecular, depending on where the structure is formed (Figure 1). Intermolecular bonds are formed by attraction between molecules. They have weak bonding due to dipole moments and dispersion forces that do not share electron pairs. Thus, the equilibrium state can be easily deformed by various external stimuli [6]. Furthermore, due to the wide range of bonding strengths, the types of bonds break randomly depending on the environmental conditions. For example, dispersion has 0.05–40 kJ/mol of bonding strength, and hydrogen bonding also has 10–40 kJ/mol. For this reason, it is difficult to determine a specific temperature range for the destruction and reformation of intermolecular bonds. Typical examples of such bonds are hydrogen bonds, ionic bonds, π–π stacking, and host–guest interactions [7,8,9]. On the other hand, intramolecular bonds are bonds in which two atoms share electron pairs with a strong bonding force. Since the intramolecular dynamic bond has an intermediate bond-dissociation energy, it is possible to reversibly dissociate and reform the bond by external stimuli (light, temperature, and pH changes) [10,11,12]. Representative intramolecular dynamic covalent bonds include the imine bond, Diels-Alder reaction, boronic/boronate ester bond, disulfide bond, and hindered-urea bond [13,14,15,16]. Dynamic covalent bonding can shift equilibrium through kinematic and thermodynamic control to determine the end product, which can improve the control and self-healing properties of materials [17,18,19,20].

Dynamic covalent bonds undergo exchange or formation reactions. In an exchange reaction, the existing reaction part is replaced by another part with the same type of bond. Ester exchange, disulfide exchange, and imine reaction correspond to this exchange reaction [21,22,23]. In a formation reaction, molecules can react to form a covalent bond. Hindered-urea bond, the Diels-Alder reaction, and aldol condensation reactions involve a formation reaction [24,25,26]. The destruction and reformation of the dynamic covalent bonding are determined by the minimum state of the thermodynamic energy [27,28]. The generated dynamic covalent bonding is thermodynamically favored in the direction in which the bonding is broken upon exposure to external stimuli, with equilibrium shifting in that direction. Conversely, when external stimuli are removed, the equilibrium shifts back toward forming bonds. Eventually, dynamic covalent bonds exhibit reversible properties [29,30].

**Figure 2:**
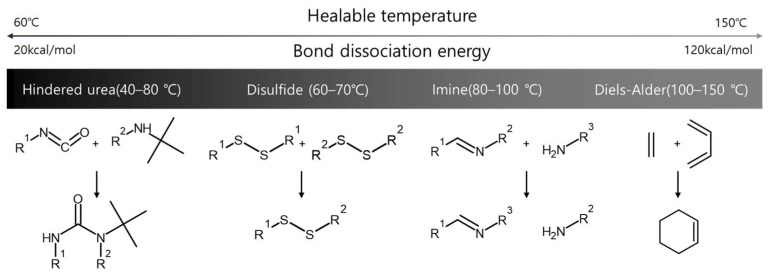
Types of dynamic covalent bonds that react reversibly to temperature changes and operating temperature ranges.

Among various external stimuli, temperature is a variable that can be easily controlled through heating and cooling processes. In particular, hindered-urea, disulfide, imine, and Diels-Alder bonds are representative bonds that show the formation and destruction of reversible bonds depending on temperature (Figure 2) [31,32,33]. The temperature range of healable properties can be explained by the bond dissociation energy [34]. Low binding energy can easily show reversible properties even under low-temperature conditions. On the other hand, since high binding energy means high strength of intermolecular bonds, a higher temperature is required to destroy and reformulate bonds [35]. This paper describes the principles, synthesis methods, properties, and applications of four representative dynamic covalent bonds operating in the intermediate temperature range. It can show the thermal response of dynamic covalent bonds and be helpful in many current and future applications, including coatings, drug delivery, smart chemical systems, and molecular devices.

## 2. Hindered-Urea Bond

### 2.1. Bonding Mechanism and Kinetic

**Figure 3:**
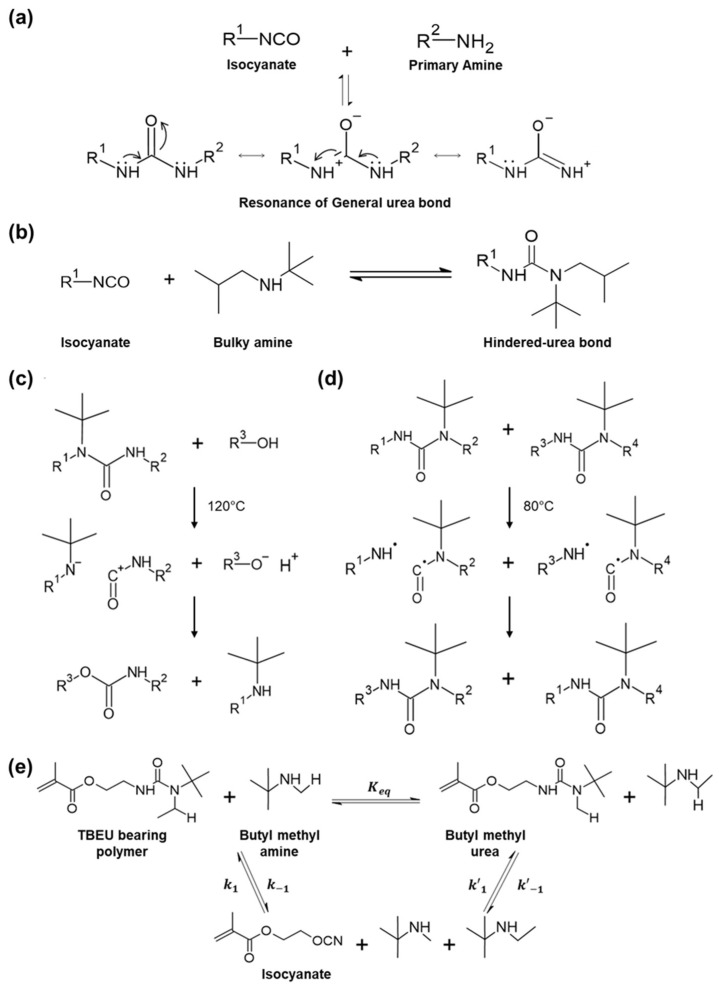
Mechanism and kinetics of hindered-urea bond. (**a**) Association–dissociation reaction of the general urea bond. (**b**) Association–dissociation reaction of the hindered-urea bond. (**c**) Heterolytic hindered-urea exchange. (**d**) Homolytic hindered-urea exchange. (**e**) Kinetics of hindered-urea bond.

The reaction between the primary amine and isocyanate makes a urea bond, which has a conjugation effect. This effect is due to covalent bonding between the lone electron pair on the nitrogen atom of the amine group and π-electrons on the carbonyl p-orbital of the isocyanate [36,37,38]. The urea product formed from a rapid reaction between the primary amine and isocyanate is highly stabilized by resonance. Consequently, a general urea bond requires extreme conditions (strong basic or acidic solution) or a catalyst for a reversible reaction (Figure 3a) [39,40,41].

The hindered-urea bond is a reaction that introduces bulky substituents into the nitrogen atom in an amine. It has about 10 kcal/mol free energy [42]. A bulky substituent makes a steric hindrance that induces bond distortion, which diminishes the conjugation effect [43,44,45,46]. As a result, the carbonyl-amine interaction is weakened, reducing the stability of the urea bond. Consequently, the hindered-urea bond can reversibly dissociate into isocyanate and amine under mild conditions, making it a notable example of a dynamic covalent bond (Figure 3b) [47,48,49,50,51].

The hindered-urea bond can undergo exchange reactions between polymer chains upon heating. The exchange reaction between chains makes it possible to reprogram the geometric and mechanical properties of the resulting network polymer [52]. The exchange reaction involves two different rearrangement mechanisms.

The first mechanism is a heterolytic hindered-urea exchange. This reaction is activated at a high temperature (120 °C), involving the disruption of the alcohol molecule bond and the breakdown of the bond between the carbonyl group and the bulky amine in the reactant containing the hindered-urea bond. As a result, a urethane bond and a hindered amine group are newly created, which are irreversible because this process consumes energy during this process (Figure 3c).

The second mechanism is homolytic hindered-urea exchange. This reaction is activated at a lower temperature (80 °C). The bond between the carbonyl group and the amine in the reactant bearing the hindered-urea bond is destroyed by the influence of the temperature. It generates two radicals each [53,54]. These radicals exchange and combine with each other, forming a new hindered-urea bond. Radicals generated in this process are relatively stable, leading to a low activation energy required for the exchange process. Additionally, since the total energy change in the bonds before and after the exchange is small, a little heat is released or absorbed during the reaction, making the thermal reaction proceed as a reversible reaction easily. Therefore, homolytic hindered-urea exchange can proceed as a reversible reaction under mild conditions. It can be effectively utilized in the transformation and synthesis of various compounds (Figure 3d).

However, bonds having bulky properties must have large dissociation and binding constants. The equilibrium must favor the formation of polymers to exhibit these dynamic and reversible properties [55]. Ying et al. explored the structures of substituents that satisfy these conditions by adjusting the volume of the substituent (Figure 3e) [36]. For example, in the case of 2,2,6,6-tetramethylpiperidinylcarboxyamide (TMPCA) with a very bulky substituent, it has a small binding constant (K_eq_ = 88), indicating a weak bond strength. However, when 1-(tert-butyl)-1-ethylurea (TBEU) is used with 2-isocyanatoethyl methacrylate, it has a large binding constant (K_eq_ = 7.9 × 10^5^), indicating a strong bond strength. In addition, when intermediate trapping experiments were performed to determine dissociation constants (k_−1_), it was confirmed that while other substituents had much smaller dissociation constants even at high temperatures, TBEU had a large dissociation constant (0.042 h^−1^) at room temperature. This confirms that efficient dynamic bond exchange is possible under mild conditions.

### 2.2. Synthesis

**Figure 4:**
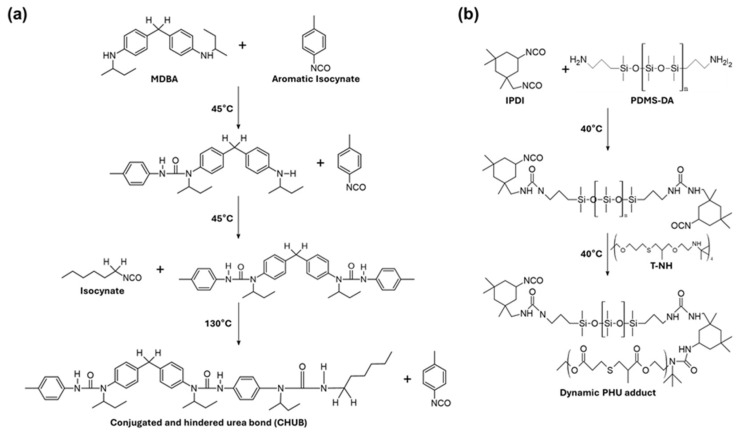
Synthesis process of dynamic hindered-urea bond. (**a**) Synthesis of conjugated and hindered-urea bond using two isocyanates with different characteristics. (**b**) Synthesis process of dynamic poly(hindered-urea) by a facile step-growth polymerization.

The hindered-urea bond can have additional abilities, such as mechanical properties, depending on substituents. Li et al. synthesized a polyurea (PU) having a conjugated and hindered-urea bond (CHUB) using 4,4′-bis(sec-butyl)methylenedianiline (MBDA) and p-tolyl isocyanate (TI) as aromatic substituents [56]. They also added hexyl isocyanate (HI) having a sec-butyl group, resulting in the coexistence of bulky and aromatic substituents (Figure 4a) [57,58]. This PU exhibits improved tensile properties and high mechanical recycling properties. Additionally, it showed excellent thermal-triggered healing properties in the coating form or in the bulk form.

Patel et al. synthesized linear adducts of (IPDI)_2_-PDMS linear adducts using bis(3-amino-propyl) terminated polydimethylsiloxane (PDMS-DA) and isoporone diisocyanate (IPDI) [59]. They then employed 4-arm star t-butylamine(T-NH) having a tert-butyl group in a step-growth polymerization through polyaddition to produce poly(hindered-urea) (PHU) (Figure 4b) [60]. This synthesis was carried out at a low temperature of 40 °C based on the sequential reaction of isocyanate and two amine monomers. The PHU has a network where t-butyl groups within hindered-urea bonds are crosslinked with amine groups from other hindered-urea bonds. When the temperature exceeded 35 °C, a bond-exchange reaction occurred in which the t-butyl group detached from the original amine and crosslinked with another amine of the same structure present in the network. This PHU exhibited recovery of mechanical properties and long-term stability after self-healing.

### 2.3. Application

**Figure 5:**
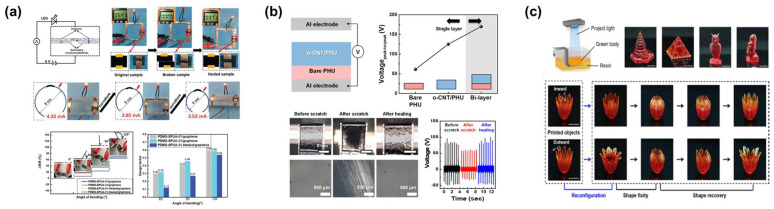
Application of dynamic hindered-urea bonded materials. (**a**) The healing process and changes in electrical properties based on the concentration of hindered-urea bonds. Reprinted/adapted with permission from Ref. [61]. 2021, Royal Society of Chemistry. (**b**) Improvement and recovery of electric properties in a bilayered polymer composite film with reversible hindered-urea bond. Reprinted/adapted with permission from Ref. [59]. 2020, American Chemical Society. (**c**) A schematic illustration of the 3D printing process and photos of 3D samples with permanent shape reconfiguration due to homolytic bond exchange. Reprinted/adapted with permission from Ref. [52]. 2023, Springer Nature.

Hindered-urea bonds have various applications, such as in electronic devices, due to their self-healing properties at low temperatures and electrical properties, depending on additives. Sun et al. synthesized a self-healing conductive polymer by introducing graphene particles, a conductive material, into PDMS containing dynamic hindered-urea bonds of various concentrations (Figure 5a) [61]. Concentrations were adjusted by controlling the amounts of piperazine and glycerol. When a voltage of 6 V was applied, the LED in the sample successfully lit up even after breaking and self-healing. Furthermore, the circuit based on healable composites maintained a conductive state despite an increase in circuit curvature. This demonstrated excellent flexibility and self-recovery ability for the electrical performance of the conductive path formed in polymers.

Patel et al. fabricated a composite film by introducing oxidized multi-walled carbon nanotubes into a material with hindered-urea exchange reactions [59]. They then used this film to create a sensor with triboelectric performance by inducing interfacial polarization (Figure 5b) [62,63]. The self-healing poly(hindered-urea) (PHU) and oxidized multi-walled carbon nanotube (o-CNT) composite were used to make an o-CNT/PHU film. It consisted of a bilayer structure, showing the enhanced output peak-to-peak voltage (169.9 V) compared to a single-layer film. The triboelectric voltage of the PHU film before self-healing decreased from 125 V to 88 V due to a decrease in the contact area through an uneven surface. However, it recovered to 130 V after healing at 90 °C. This demonstrated that the film had high recoverability and excellent mechanical strength [64].

Fang et al. utilized a homolytic hindered-urea exchange rearrangement to create a material named HUBM-co-PPGA by introducing poly(propylene glycol) (PPGA) as hydroxyl groups into hindered-urea containing bismethacrylate (HUBM) (Figure 5c) [52]. Networks of HUBM-co-PPGA were synthesized by light-mediated free-radical polymerization in the presence of a photo-initiator (Irgacure 319). Using the digital light processing (DLP) liquid printing precursor made by HUBM-co-PPGA, they fabricated flower-shaped 3D samples having shape reprogrammability characteristics by repetitive and sequential rearrangement under 80 °C and external deformation force. The resulting 3D samples enabled permanent shape reconfiguration at 80 °C and exhibited shape memory behaviors with shape fixing and recovery between −40 and 60 °C. All these shapes remained thermodynamically stable even when heated to 60 °C. Moreover, these newly created permanent shapes could be manipulated by heating above the deformation temperature (glass transition temperature). This was because the homolytic exchange reaction activated at 80 °C had a macroscopic shape reprogramming function through plasticity [65,66,67].

## 3. Disulfide Bond

### 3.1. Bonding Mechanism and Kinetic

**Figure 6:**
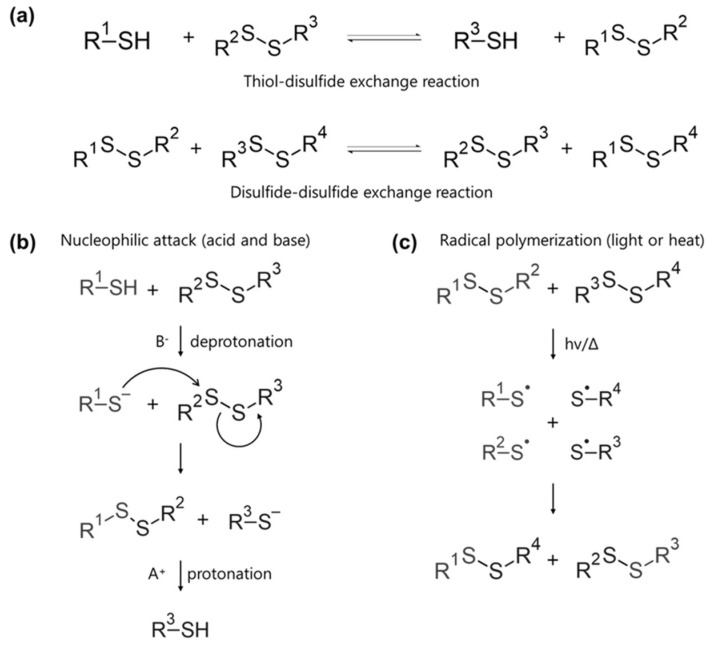
Dynamic disulfide bonding mechanism and structures. (**a**) Thiol-disulfide and disulfide-disulfide exchange reactions. (**b**) Nucleophilic attack mechanism with acid and base. (**c**) Radical polymerization induced by light or heat.

Disulfide bonds are among the most used dynamic bonds, typically with a dissociation energy of 60 kcal/mol [68]. Compared to selenides from the same group 16, sulfur forms more stable bonds, which leads to excellent self-healing abilities and diverse applications [69]. Dynamic disulfide bonds are formed through thiol-disulfide exchange reactions and disulfide-disulfide exchange reactions (Figure 6a) [70,71].

The thiol-disulfide exchange reaction involves a nucleophilic attack in the presence of acids or bases (Figure 6b) [72]. Initially, thiol is deprotonated to generate a thiolate anion, which then breaks the S-S bond and forms a new bond. The resulting thiolate anion is subsequently protonated back to thiol. In contrast, disulfide exchange reactions can also proceed via radical polymerization mediated by light or heat (Figure 6c) [73]. Light or heat induces the formation of radicals, which decompose the disulfide bond. During the propagation stage, the radical acceptor binds the activation site to terminate the reaction. In conventional free radical polymerization, it is difficult to control the time of the propagation chain, often leading to non-uniform polymers. To address this, methods such as atom transfer radical polymerization (ATRP), single-electron transfer-living radical polymerization (SET-LRP), nitroxide-mediated polymerization (NMP), and reversible-addition-fragmentation chain-transfer (RAFT) polymerization have been introduced to create polymers with predictable and accurate molecular weight and physical properties [74,75,76,77].

A nucleophilic attack by a chemical trigger does not require external stimuli such as temperature or light. It can be easily used to synthesize various thiol-containing materials. However, it is sensitive to pKa. It has a slow reaction rate compared to radical polymerization [78]. On the other hand, radical polymerization is fast, and it can use a variety of monomers [79]. Further research is needed on the selectivity of the controlled reaction.

**Figure 7:**
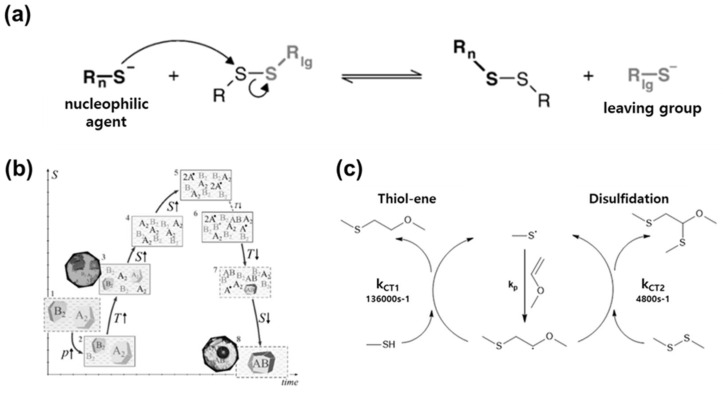
Kinetics of dynamic disulfide bond. (**a**) Disulfide exchange reaction based on S_N_2 mechanism. Reprinted/adapted with permission from Ref. [83]. 2007, Elsevier. (**b**) Schematic diagram illustrating changes in thermodynamic states according to disulfide bond formation. Reprinted/adapted with permission from Ref. [85]. 2021, American Chemical Society. (**c**) Comparison of reaction pathways and equilibrium constants for thiol-ene and disulfide-disulfide reactions. Reprinted/adapted with permission from Ref. [86]. 2022, American Chemical Society.

Dynamic properties of disulfides are determined by basic kinetic mechanisms and various parameters [80,81,82]. In particular, the disulfide exchange reaction through the S_N_2 reaction largely depends on the pKa and nucleophilicity of the thiolate, which can affect the reactivity of the thiol (Figure 7a) [83,84]. A highly nucleophilic thiolate facilitates the disulfide exchange reaction. As the pKa increases, the nucleophilicity of the thiolate also increases, accelerating the reaction. Moreover, central sulfur with a stable leaving group and a high electron affinity lowers the activation barrier.

Additionally, dynamic bonds of disulfide are formed and cleaved according to their thermodynamic properties (Figure 7b) [85]. Cleavage of a homodimer can increase entropy and generate radicals, which subsequently form heterodimers. Crystallization of the resulting product then decreases entropy, leading to thermodynamic equilibrium. The equilibrium constant (K) at this point is influenced by thermodynamic factors such as temperature, pressure, solvent, and concentration.

Macroscopically, thiol-disulfide and disulfide-disulfide reactions exhibit distinct mechanical differences. The equilibrium constant (k_ct1_) for the thiol-ene reaction is 136,000 s^−1^, while the equilibrium constant (k_ct2_) for the disulfide reaction is 4,800 s^−1^ [86]. Thus, the thiol-ene reaction is approximately 30 times faster than the disulfide reaction (Figure 7c). This is because greater steric hindrance occurs during the chain transfer step of the disulfide-disulfide reaction. A comprehensive understanding of the kinetics and thermodynamics of disulfide reactions can facilitate the effective design of materials.

### 3.2. Synthesis

**Figure 8:**
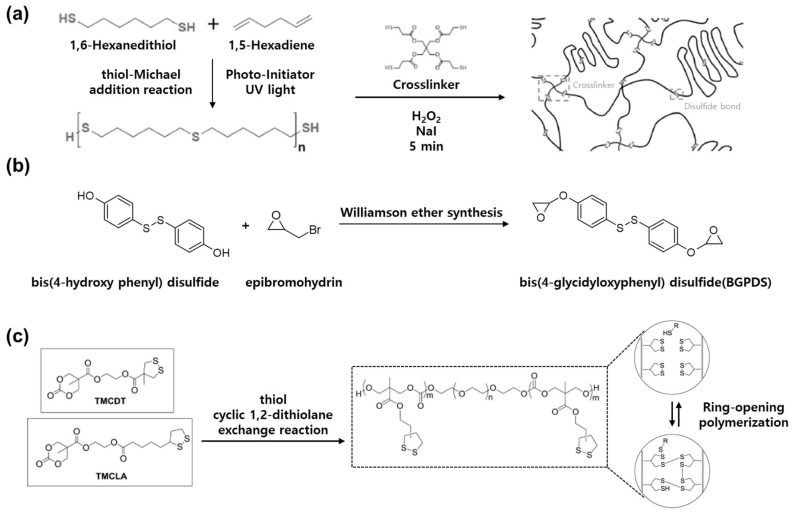
Synthesis of dynamic disulfide bonds. (**a**) Synthesis of a polydisulfide network based on the thiol–Michael reaction between thiol and alkene. Reprinted/adapted with permission from Ref. [87]. 2023, American Chemical Society. (**b**) Synthesis of degradable epoxy resin via the Williamson ether synthesis method. Reprinted/adapted with permission from Ref. [88]. 2016, Elsevier. (**c**) Synthesis of copolymers containing disulfide networks using cyclic 1,2-dithiolane. Reprinted/adapted with permission from Ref. [89]. 2017, American Chemical Society.

Dynamic disulfide bonds can be integrated into polymers using various synthesis methods. Nguyen et al. fabricated a polydisulfide network (PDSN) containing reversible disulfide bonds using pentaerythritol tetrakis (3-mercapto propionate) (Figure 8a) [87]. Initially, a biothiol oligomer was synthesized using hexadiene and hexanedithiol. Subsequently, oxidizing agents such as sodium iodide and hydrogen peroxide were used to remove hydrogen from thiol groups, creating activation sites for disulfide bond formation. This method utilizes the thiol–Michael reaction to synthesize a thioester through the reaction between a thiol and an alkene.

Takahashi et al. synthesized bis(4-glycidyloxyphenyl)disulfide (BGPDS) using bis(4-hydroxyphenyl)disulfide and epibromohydrin (Figure 8b) based on the Williamson ether method, which synthesizes ether from organic halides and alkoxides [88]. This creates diepoxdie moieties that include disulfide bonds, allowing the polymer to split into smaller fragments after decomposition. As a result, BGPDS achieved an effective disulfide exchange reaction with diphenyldisulfide in the presence of a base.

Zhang et al. prepared a dynamic covalent material using the reaction between thiol and cyclic 1,2-dithiolane, which provides a fast exchange rate with reversibility (Figure 8c) [89]. First, they selected two monomers, TMCDT and TMCLA, with different structures and substitution patterns. The ABA copolymer made through ring-opening polymerization consists of a central poly(ethylene oxide) block and a terminal polycarbonate block. The copolymer exhibits amphiphilic properties and self-assembles into flower-like micelles at high concentrations.

When thiol was added to the material, the core containing disulfide bonds rapidly underwent an exchange reaction and formed a temporary network. Macroscopic properties such as thermal stability and elasticity varied depending on the type and ratio of monomers. As a result, they demonstrated the synthesis of effective dynamic disulfide materials through analysis of the dynamics and behavior characteristics of 1,2-dithiolane-based polymers.

### 3.3. Application

**Figure 9:**
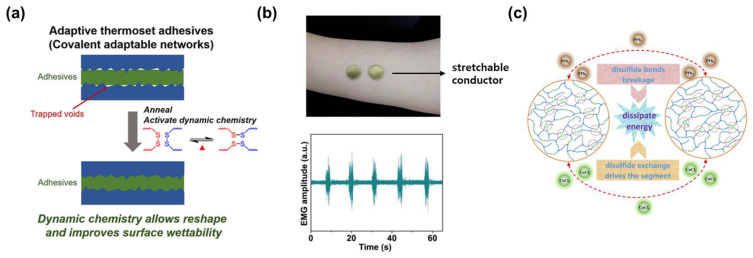
Application of dynamic disulfide bonded materials. (**a**) Improvement in wettability in adaptive thermosetting adhesives through the introduction of dynamic disulfide bonds. Reprinted/adapted with permission from Ref. [94]. 2020, American Chemical Society. (**b**) Polyurethane-based stretchable conductors exhibiting low hysteresis characteristics under strain. Reprinted/adapted with permission from Ref. [95]. 2022, Royal Society of Chemistry. (**c**) Damping material based on the absorption of energy upon release of bonding in aromatic disulfides. Reprinted/adapted with permission from Ref. [96]. 2021, Wiley.

Disulfide bonds can improve the properties of materials and expand their applications through their self-recovery and reprocessing ability, strong mechanical strength, and high stretchability [90,91,92,93]. For instance, shrinkage during the curing process of thermosetting materials has a negative effect on adhesive performance. To address this issue, Li et al. manufactured an adaptive thermosetting adhesive with improved properties by introducing disulfide bonds into the polymer (Figure 9a) [94]. Adhesion performance increased approximately four times due to the dynamic change and reconstruction of the network through the disulfide bond. In this way, the performance was improved by applying dynamic disulfide bonding to the adhesive material.

Disulfide bonding also enables the fabrication of stretchable self-healing materials. Kong et al. incorporated disulfide bonds to create a stretchable conductor that can resist mechanical damage (Figure 9b) [95]. The conductor consisted of a thin gold film deposited on a polyurethane-based polymer substrate containing disulfide bonds. Remarkably, this conductor exhibited ultra-low hysteresis properties, with a strain value of 812% at room temperature and 3.8% at 100% strain. After cutting, it effectively restored its electrical properties to near-original levels, demonstrating excellent elastic self-healing material behavior. This stretchable conductor performed exceptionally well as an electrode in electromyography (EMG) monitoring.

Aromatic disulfide compounds exhibit exchange reactions at room temperature, absorbing energy during bond formation or cleavage. Ma et al. employed a dynamic aromatic disulfide system to create an attenuation material functional at room temperature (Figure 9c) [96]. When a dynamic aromatic disulfide bond breaks, it absorbs external energy, such as vibration and sound. Upon reformation, it efficiently dissipates energy by promoting segment movement and rearrangement. Polyurethane incorporating this disulfide system exhibited an impressive damping range from 48 °C to 100 °C. It also showed a tensile strength of 16.8 MPa and an elongation at a break of 926%. The strategic incorporation of disulfide bonds into various materials and systems shows potential for improving the self-healing properties and elasticity of next-generation materials.

## 4. Imine Bond

### 4.1. Bonding Mechanism and Kinetic

**Figure 10:**
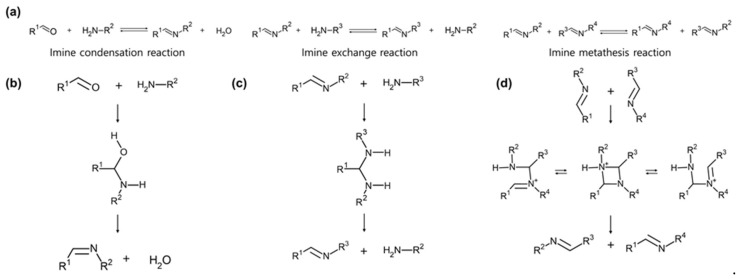
Dynamic imine bond mechanisms and structures. (**a**) Three types of imine bond formation and dissociation. (**b**) Mechanism of imine condensation reaction. (**c**) Mechanism of imine exchange reaction. (**d**) Mechanism of imine metathesis reaction.

Imine bonds formed from amines and aldehydes are among the oldest and most prevalent reactions in organic chemistry [97]. These bonds feature a double bond between carbon and nitrogen, comprising two alkyl or aryl groups and a Schiff base with a single hydrogen [98]. The bond dissociation energy of the imine bond depends on the alkyl group attached to the double bond of carbon and nitrogen [99]. It ranges from approximately 80 to 100 kcal/mol. Accordingly, the self-healing temperature of the imine bond appears to be around 80 to 100 °C [100,101,102]. Reactions of imine bonds can be categorized into condensation reactions, exchange reactions, and metathesis reactions (Figure 10a) [103].

The imine condensation reaction occurs through a hydrolysis process where an amine and an aldehyde react, releasing water and forming an imine (Figure 10b) [104]. Therefore, when water is added to the imine product, the equilibrium shifts toward the original product according to Le Châtelier’s principle, and reversibility can be achieved [105]. In the case of an imine exchange reaction, the two protons of the amine move to the nitrogen atom of the imine (Figure 10c) [106]. When a second amine is introduced into this reaction, the original imine undergoes transimination and the alkyl group can be exchanged. Finally, the imine metathesis reaction involves a scrambling reaction between two preformed imines (Figure 10d) [107]. The process involves a four-membered cyclic transition state in which imine bonds are exchanged.

**Figure 11:**
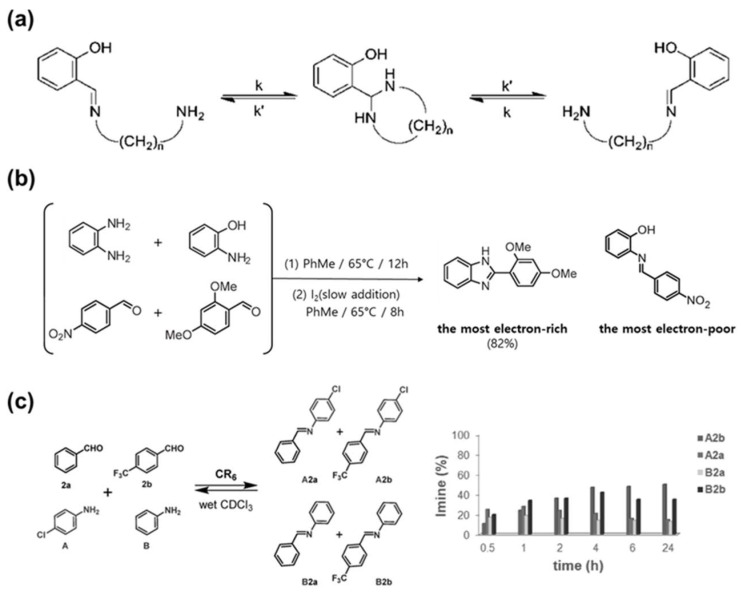
Kinetics of the dynamic imine bond. (**a**) Local molecular motions involving the imine exchange reaction. Reprinted/adapted with permission from Ref. [112]. 2012, American Chemical Society. (**b**) [2 × 2] dynamic combinatorial library of imine bonds composed of amines and aldehydes and its thermodynamic products. Reprinted/adapted with permission from Ref. [113]. 2011, American Chemical Society. (**c**) Kinetic and thermodynamic products of the dynamic covalent library in the presence of hexameric resorcinarene capsules. Reprinted/adapted with permission from Ref. [19]. 2020, American Chemical Society.

The formation and exchange of imine bonds are influenced by several factors, including solvent, concentration, pH, temperature, steric effects, and electronic effects [108,109,110,111]. Kovaříček et al. conducted a dynamic analysis of the influence of certain parameters on the formation and exchange of imine (Figure 11a) [112]. In the case of dialiphatic diamine forms of mono imines, local motion occurs, leading to bond exchanges within the molecule. The rate of this exchange is regulated by substituents, solvent composition, and temperature. Strong electron-withdrawing substituents can enhance dynamic imine reactions by increasing bond polarity. Furthermore, temperature variations can further modulate this reactivity, allowing precise control of reaction kinetics. Additionally, as solvent polarity increases, the exchange rate of dynamic bonds decreases.

Osowska et al. investigated the kinetics and thermodynamics of an [*n* × *n*] dynamic library composed of amines and aldehydes (Figure 11b) [113]. As a result of the slow and irreversible oxidation process, this library is simplified. It yielded only *n* products because the imine formation and exchange occur preferentially in an electron-rich environment. It leads to the presence of two primary imine types in the final product: the most electron-rich and the most electron-poor imine.

Along with other dynamic bonds, imine bonds can result in the replacement of competing intermediates with the thermodynamically most stable product over time. However, the composition of the dynamic combinatorial library (DCL) can change depending on external stimuli, which also affects the final product. Gambareo et al. analyzed the kinetic and thermodynamic modulation of the imine component in the presence of a hexameric resorcinarene capsule (Figure 11c) [19]. In general, imines formed immediately after the reaction showed a decrease in kinetic products (A2a, B2b) and an increase in thermodynamic products (B2a, A2b) over time. External stimuli can promote the formation of specific products and influence the amount of increase or decrease in products. Understanding the kinetics of the imine library and the influence of external stimuli can contribute to the effective implementation of materials design.

### 4.2. Synthesis

**Figure 12:**
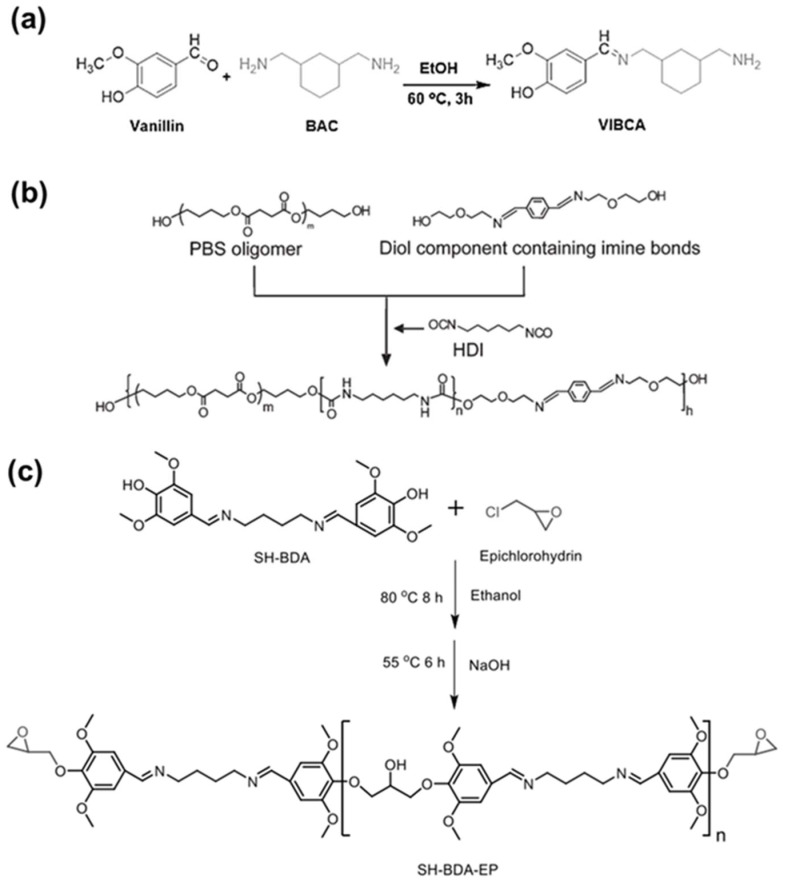
Synthesis of dynamic imine bond. (**a**) Synthesis of a vanillin-based curing agent via a single imine condensation reaction. Reprinted/adapted with permission from Ref. [15]. 2022, American Chemical Society. (**b**) Synthesis of a green dynamer from biodegradable oligomer and diol component containing imine bonds. Reprinted/adapted with permission from Ref. [114]. 2012, Royal Society of Chemistry. (**c**) Synthesis process of thermosetting epoxy incorporating Schiff base chemistry. Reprinted/adapted with permission from Ref. [115]. 2021, Elsevier.

Imine bonds are used in various synthetic methods because they can perform various reversible reactions, such as imine condensation and transimination, without a catalyst. In particular, the imine condensation reaction can be completed through a single-step process, making it ideal for a simple synthesis method. Rashid et al. synthesized a vanillin-based imine curing agent (VIBCA) through a single-step, reversible condensation reaction of vanillin and bisaminomethyl cyclohexane (BAC) (Figure 12a) [15]. An imine bond was formed through a Schiff base reaction using the aldehyde group of vanillin and the amine group of BAC. The imine bond is implemented through this simple and effective synthetic process, triggering a dynamic reaction, and giving the material the ability to recover and recycle.

Fukuda et al. developed a degradable polymer by linking polybutylene adipate (PBA) and polybutylene succinate (PBS) through an imine bond [114]. The diol component containing an imine bond was prepared by a reaction of terephthaladehyde and 2-(2-aminoethoxy)ethanol. Subsequently, this bisimine was synthesized into the final polymer through a condensation reaction with the biodegradable oligomer PBS and hexamethylenedisocyanate (HDI) (Figure 12b). The dynamic polymer effectively achieved the reversibility of imine bonding in the presence of water.

Schiff base chemistry is a useful method to synthesize imines that provide bond dynamics, self-crosslinking properties, and excellent mechanical properties. The self-crosslinking property of Schiff base polymers involves the rearrangement and cyclization of imine bonds in the main chain at high temperatures, leading to the formation of a nitrogen-containing 6-membered heterocyclic compound and the subsequent creation of a stable network. Xie et al. synthesized imine-based materials with various functionalities based on Schiff base chemistry [115]. They prepared SH-BDA, containing an imine bond by reacting syringaldehyde (SH) with 1,4-butanediamine (BDA). Additionally, they synthesized SH-BDA-EP by mixing SH-BDA with epichlorohydrin (ECH) in the presence of tetrabutylammonium bromide (TBAB) (Figure 12c). The synthesized imine-based polymer was finally manufactured into a bio-epoxy thermoset material through reaction with diaminodiphenylmethane (DDM), a curing agent.

### 4.3. Application

**Figure 13:**
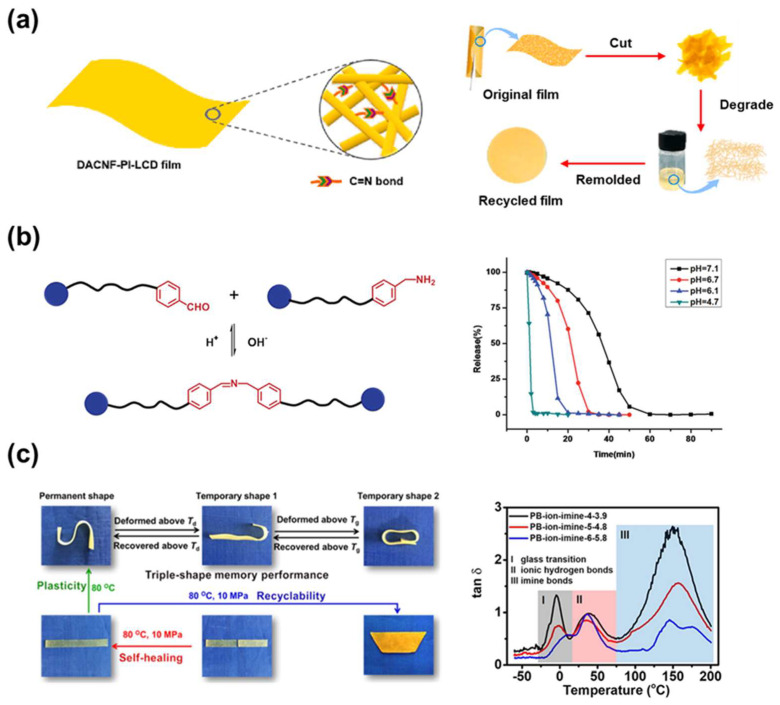
Application of dynamic imine bonded materials. (**a**) Fabrication of biodegradable polymers utilizing imine bonds and cellulose nanofibers-. Reprinted/adapted with permission from Ref. [116]. 2023, Elsevier. (**b**) Development of a pH-sensitive amphiphilic bolaform driven by benzoic imine bonds. Reprinted/adapted with permission from Ref. [117]. 2011, American Chemical Society. (**c**) Implementation of a dual-crosslinked polymer network with multi-shape memory through multiple thermal transition states. Reprinted/adapted with permission from Ref. [118]. 2020, American Chemical Society.

Imine bonds are used in a variety of applications due to their facile bond formation process and reversible dynamic properties. In particular, imine bonds can be used as crosslinks for materials to improve the mechanical properties of polymers. Zhang et al. fabricated a biodegradable polymer incorporating imine bonds derived from biomaterials to solve problems of petroleum-based plastics (Figure 13a) [116]. Reversible imine bonds were formed through dialdehyde groups of cellulose nanofibers and plant oil-based aliphatic diamine monomers. Dynamic crosslinking through imine bonds provided excellent mechanical strength and thermal adaptability. The optimization of conditions was achieved by controlling the carbon chain length of the diamine monomer in the process. Additionally, dynamic imine bonds enabled reversible network formation, degradability, and reformation of polymer films after hot pressing at 1.5 MP for 15 min. In this way, the introduction of bio-based materials and imine bonds simultaneously produced environmentally friendly functional materials and improved mechanical properties.

Wang et al. designed a pH-sensitive amphiphilic bolaform utilizing benzoic imine bonds (Figure 13b) [117]. By incorporating dynamic covalent bonds into superamphiphiles, they achieved diverse properties beyond traditional covalent bond-based amphiphiles. In an alkaline environment, the bond remains stable. However, in a slightly acidic environment, the bond dissociates through hydrolysis. Hence, it operates as a pH-responsive assembly. Disassembly of micellar aggregates leads to the release of loaded guest molecules, which enables targeted drug delivery in a controlled biological environment. Such pH-responsive properties of imine bonds lead to effective and controllable biotechnology applications.

Zhang et al. developed a double crosslinked polybutadiene network featuring three distinct thermal transition states by combining imine bonds and ionic hydrogen bonds (Figure 13c) [118]. The incorporation of multiple thermal transition states enables the polymer to exhibit various shape memory capabilities, including multi-shape memory. Imine bonds, which have a higher activation energy than ionic hydrogen bonds, determine the permanent shape of the polymer. Meanwhile, the glass transition temperature of the polymer and the temperature associated with ionic hydrogen bonding can influence its temporary shape. This dynamic bonding enables solid-state plasticity. Notably, the polybutadiene network surpasses existing shape memory polymers by maintaining elasticity while achieving plasticity through a triple shape memory effect. The synergistic combination of imine bonds and other dynamic bonds provides various possibilities beyond the limitations of conventional polymers.

## 5. Diels-Alder Bond

### 5.1. Bonding Mechanism and Kinetic

**Figure 14:**
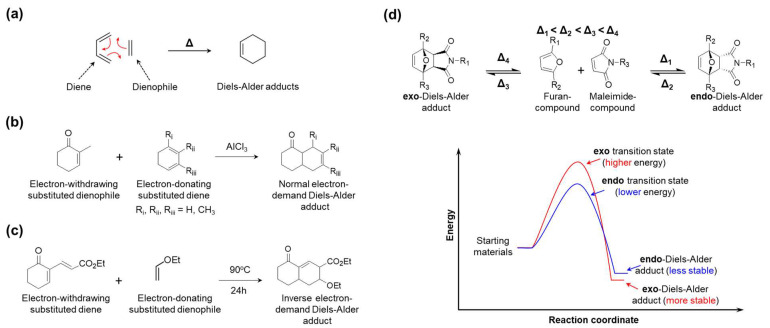
(**a**) The most basic DA reaction. (**b**) Normal electron-demand DA reactions, with an electron-poor dienophile and an electron-rich diene. (**c**) Inverse electron-demand DA reaction, with an electron-poor diene and an electron-rich dienophile. (**d**) *Endo*- and *exo*-diastereomer are obtained and lost according to DA reaction and retro DA reaction, respectively, and the energy profile diagram for its transformation.

Initially, the Diels-Alder (DA) reaction was suggested as a method to synthesize cyclic molecules and thermostable polymers [119]. Over the past few decades, this reaction has been considered not only for its molecular stabilization but also for its reversibility under thermal exposure (the retro-DA reaction), opening up a new prospect for preparing smart polymers [120,121]. In terms of the mechanism, the DA reaction is based on a powerful strategy in organic chemistry called cycloaddition [4+2], which is the reaction between a diene and a dienophile (Figure 14a). Moreover, attaching electron-donating (EDG) or electron-withdrawing (EWG) groups to the diene or dienophile could accelerate cycloaddition, known as the electronic effect [122]. From there, DA reactions are classified into two main cases.

First, the combination of electron-donating substituted dienes and electron-withdrawing substituted dienophiles is called the normal electron-demand DA reaction [123]. It is the most popular. As reported by Fringuelli et al., ketone groups (=O) work as EWG, which pull electrons out of the π system (Figure 14b). Meanwhile, methyl (–CH_3_) groups work as EDG which pushes electrons into the π system.

Second, the combination of electron-withdrawing substituted dienes and electron-donating substituted dienophiles is called the inverse electron-demand DA reaction [124]. For example, Bodwell et al. suggested the DA reaction with ethoxycarbonyl (–CO_2_Et) and ketone (=O) groups as EWG on the diene and ethoxy (–OEt) groups as EDG on the dienophile (Figure 14c).

In short, the nature of the diene/dienophile couple is essential for promoting a DA reaction. It influences reaction success. Therefore, many dienes and dienophiles have been studied. They are also well-collected in Fringuelli’s and Taticchi’s books [125,126]. In addition, interest in bio-based building blocks is growing nowadays, leading to obtaining new bio-derived dienes and dienophiles such as sorbic acid, myrcene, 3-hydroxy-2-pyrone, citraconic acid, itaconic acid, and vinyl ketones [127,128,129,130,131,132].

Furthermore, by subjecting to kinetic and thermodynamic controls, the DA reaction becomes a member of the reversible covalent bond community. In the last decade, Froidevaux et al. conducted an insight study to establish precise rules of furan and maleimide derivative substituents that could influence obtaining DA diastereomers (*endo* and *exo* adducts) and their reversibility (Figure 14d) [133]. It is clear that the energy needed for the *endo*-transition state (Δ_1_) is lower than that for the *exo*-transition state (Δ_3_). In terms of kinetic control, the DA reaction has to be carried out at a low temperature to increase the *endo* adduct. Moreover, electron-withdrawing R_2_ and R_3_ substituents and electron-donating R1 substituents also contributed to favoring *endo* adducts. On the other hand, the *endo* adduct is unblocked at a lower temperature than the *exo* one (Δ_2_ < Δ_4_), which is thermodynamically more stable. In terms of thermodynamic control, with the support of electron-withdrawing R_2_ and electron-attracting mesomeric R_1_ substituents in the adducts structure, the unblocking reaction rate is faster. Based on this phenomenon, the range of DA/retro-DA temperatures of various other diene/dienophile couples can be expanded and be approachable for more applications.

### 5.2. Synthesis

In the field of synthesizing self-healable DA-contained polymers, a diverse range of diene/dienophile couples have been suggested [134]. Strategies to introduce DA bonds in the polymer structures can be categorized into three categories: (1) diene/dienophile-crosslinkers; (2) DA-contained monomers/oligomers; and (3) monomers bearing diene/dienophile couples.

**Figure 15:**
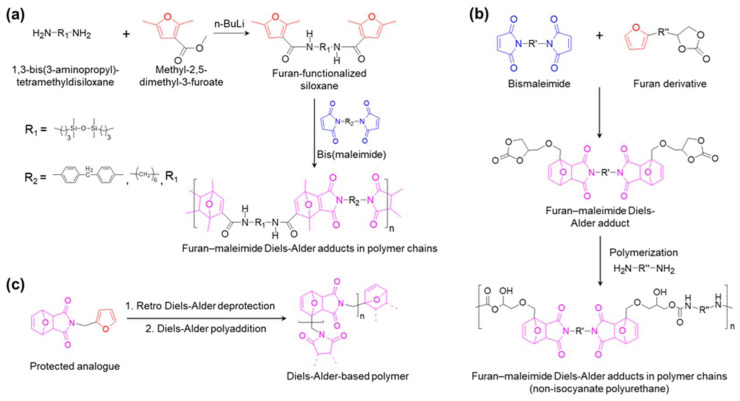
Three strategies to introduce DA bonds into the polymer structures: (**a**) using diene/dienophile-crosslinkers; (**b**) using DA-contained monomer/oligomer; and (**c**) using monomers bearing diene/dienophile couples.

The first strategy suggested by Tesoro and Sastri consists of functionalizing one or more units of the polymer with furan or maleimide moieties (Figure 15a) [135]. Polyaddition between furan-functionalized polymer and maleimide-functionalized polymer was then conducted to obtain linear DA bond-incorporated polymer chains. In detail, methyl-2,5-dimethyl-3-furoate underwent an amidation reaction with l,3-bis(3-aminopropyl)-tetramethyldisiloxane to generate furan-functionalized siloxane. It was then polymerized with bis(maleimides) in THF at 70 °C. In approaching strategy (1), plenty of different diene/dienophile-functionalized monomers were designed, which were not only diverse in diene/dienophile substituents, but also various in the type of polymer chain such as linear structure, branched (block copolymer), crosslinked, and network structures [136]. For example, Jiang and Hadjichristidis utilized this strategy to incorporate receptor-substituted luminogens into polymer networks using dimaleimide-substituted tetraphenylethene as a crosslinker [137].

Strategy (2) consists of developing DA bond-incorporated polymers using the DA reaction between monomers, with one of its terminals modified with furan and another modified with maleimide groups (Figure 15b). Elena Dolci et al. suggested this strategy to synthesize reversible non-isocyanate polyurethanes to restrict the harm of diisocyanates [138]. In detail, the DA dicyclocarbonate adducts as monomers were synthesized through a DA reaction between furfuryl alcohol and bismaleimide polymer. The non-isocyanate polyurethane was then obtained after step-growth polymerization of monomers with diamines. However, the retro-DA reaction can significantly restrict the success of the synthesis following this pathway. Thus, the adduct polymerization should take place at room temperature even though it will slow down the rate of cyclocarbonate/amine reactions. Thus, triazabicyclodecene as a catalyst was introduced to accelerate the reaction. Furthermore, it is possible to directly functionalize a DA adduct to obtain the monomer of interest, such as a polyol for the synthesis of polyester or some epoxy prepolymer [139,140].

Regarding strategy (3), a monomer was functionalized to bear both furan and maleimide end-groups (Figure 15c). To prevent early side-polymerization, protection–deprotection steps must be followed during the preparation of these monomers. For example, Lacerda et al. devised a simple furan-maleimide AB-type monomer containing a single methylene moiety bridging the furan heterocycle with the maleimide counterpart [141]. At the initial step, 2-furfurylamine and a previously synthesized DA adduct (*exo*) reacted at about 60 °C to obtain the protected analog. Its DA adducts were then unblocked and the protecting furan (retro-DA reaction) was released at 110 °C (higher energy) in 1,1,2,2-tetrachloroethane. The DA polyaddition was activated when the system temperature was decreased back to 60 °C. Hence, it shifted the DA equilibrium towards the formation of adducts (both *endo* and *exo* stereoisomers) in the polymer backbone. Based on this study, the range of DA/retro-DA temperatures of various other diene/dienophile couples can be expanded for more applications.

### 5.3. Application

Both academic and industrial research have applied DA dynamic bonding, especially for packaging, coating, and biomedical materials [142,143,144,145]. These bonds can facilitate the design of self-healing materials by forming and breaking covalent bonds upon exposure to temperature. The associative exchange mechanism allows for easy network reconfiguration while maintaining integrity.

**Figure 16:**
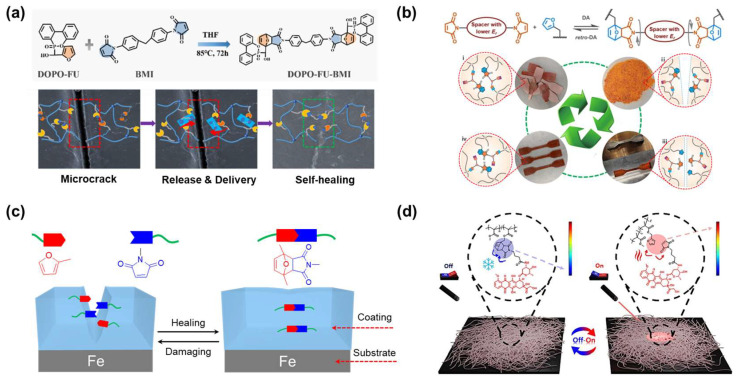
(**a**) Preparation of temperature-mediated Diels-Alder (DA) adducts and the epoxy thermoset crack–healing mechanism. Reprinted/adapted with permission from Ref. [146]. 2023, American Chemical Society. (**b**) The design concept of DA cycloadditions with lower energy barrier and the reprocess ability of the material. Reprinted/adapted with permission from Ref. [147]. 2023, Wiley. (**c**) Self-healing mechanism of the urushiol-based coating surface [150]. (**d**) A schematic illustration of the on/off switch for the drug release mechanism from nanofibers using near-infrared (NIR) light. Reprinted/adapted with permission from Ref. [153]. 2024, Elsevier.

For example, Lian et al. suggested a multifunctional DA release-delivery system that can repair epoxy thermoset crack defects (Figure 16a) [146]. A composite was fabricated through the DA reaction between the furan diene-containing phosphorus derivative (DOPO-FU) and the maleimide dienophile (BMI). It was then linked to the epoxy resin by the grafting reaction. When the damaged sample was treated at 150 °C, DA additives demonstrated their effective capacity for self-healing right away. Once the heat was turned off, maleimide dienophiles reassembled the furan groups located near the cracks and then completed the repair process. This research combines flame retardant properties, high flexural strength, and reversibility in a material that unlocks high potential for electronics packaging applications.

Gu et al. proposed a similar approach in a study on recycled composites (Figure 16b) [147]. In their study, phosphorus-based units were not only utilized as a flame-retardant factor but also as a spacer, which could accelerate the DA exchange reaction. The collaboration of trimethyl phosphate and diene/dienophile couples resulted in phosphate-derived DA cycloadditions, which provided high-efficiency repairing, reprocessing, and the rapid nondestructive recycling of carbon fibers. Their study provides insights into the molecular connotation of DA chemistry. It also provides a premise for developing reusable materials.

In addition, DA dynamic bonds have become common in the development of polymeric coatings due to their thermal reversibility, mild reaction conditions, and flexible preparation methods [148,149]. Although this material has a high self-healing efficiency, it often comes with low mechanical strength and poor structural durability. To address this challenge, Zhao et al. used the rigid benzene ring and flexible unsaturated long-side carbon chain of urushiol (as a renewable source) combined with DA dynamic bonding to generate efficient self-healing coating materials (Figure 16c) [150]. The material obtained from the combination of hard segments (benzene rings), soft segments (urushiol diglycidyl ether functionalized by furan alcohol), and crosslinkers (dimaleimide) exhibited excellent physico-mechanical and self-healing properties. Moreover, due to a greener and more sustainable demand for polymer coating, a variety of strategies using bio-based materials combined with dynamic covalent bonds have been proposed and investigated [151,152].

In drug development, DA adducts are often used as controllable linkers. Instead of depending on time or physiological activity, the use of controllable linkers allows for monitoring of payload release in vivo. For example, Scancar et al. designed a drug delivery system based on photothermally active graphene-based nanofibers containing DA bonds (Figure 16d) [153]. At mild temperatures, the DA reaction happened between pendant furan groups incorporating nanofibers and maleimide-containing drug molecules. During exposure to NIR irradiation (increasing the local temperature), the DA covalent bonds released drug molecules from the nanofibers. Consequently, a controlled release of the drug was achieved, thereby reducing passive drug release. Nanofibers obtained by this method have great potential to act as a delivery device for photothermal therapeutic molecules in the future.

## 6. Others

### 6.1. Acylhydrazone Bond

**Figure 17:**
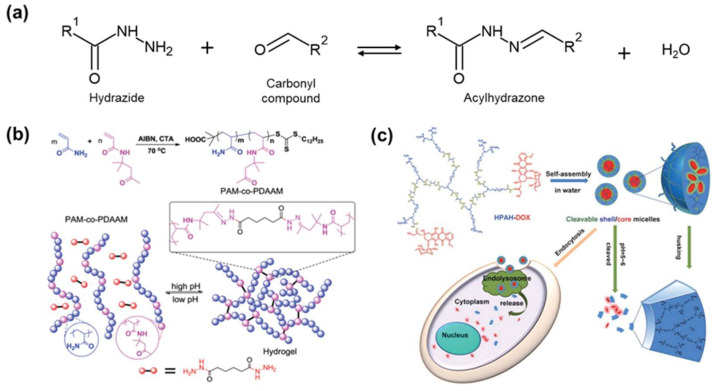
Acylhydrazone bond. (**a**) Structure and mechanism of the acylhydrazone bond. (**b**) Synthesis of ketone-based polymers via reversible addition–fragmentation chain-transfer polymerization(RAFT) polymerization and acylhydrazone crosslinking. Reprinted/adapted with permission from Ref. [158]. 2017. Royal Society of Chemistry. (**c**) Doxorubicin (DOX)-delivery system utilizing acylhydrazone bond, which is capable of releasing drugs in response to acidic conditions. Reprinted/adapted with permission from Ref. [160]. 2011, Royal Society of Chemistry.

Acylhydrazone bonds are formed through Schiff base reactions similar to imine bonds [154]. These bonds form through the condensation reaction between carbonyl and hydrazide, with water as a byproduct (Figure 17a) [155]. The reversible formation and release of acylhydrazone bonds can be controlled by adjusting the pH [156].

Polymers containing dynamic acylhydrazones are mainly synthesized through carbodiimide chemistry using polymers containing carboxyl groups and dihydrazide compounds [157]. Guo et al. fabricated a reversible stimulus-responsive hydrogel using dynamic acylhydrazone linkage (Figure 17b) [158]. A ketone-based polymer was prepared by RAFT polymerization of acylamide (AM) and diacetone acrylamide (DAAM), followed by the addition of hexanedihydrazide to create the hydrogel. Hexanedihydrazide provided two hydrazide groups to crosslink the polymer chains. The formation of acylhydrazone bonds was demonstrated by the disappearance of the C=O band of the ketone-based polymer and the appearance of a new C=N band in infrared(IR) analysis.

Acylhydrazone bonds are not only sensitive to pH but also useful in drug design and medicinal chemistry due to the drug action of the –CO–NH–N= group [159]. Zhu et al. implemented a drug delivery system using hyperbranched polyacylhydrazone (HPAH), which is cleaved in response to pH (Figure 17c) [160]. The acylhydrazone terminal of HPAH formed HPAH-DOX by combining with the anticancer drug doxorubicin (DOX). In water, HPAH-DOX self-assembles into shell/core micelles. These micelles remained stable at physiological pH 7.4, but degraded in an acidic lysosomal environment, releasing DOX for targeted drug delivery. Additionally, HPAH carriers were broken down into small molecules within the body due to their biodegradability. Dynamic acylhydrazone bonds activated by pH are effective in medical and biology fields. These are also utilized in solar cells and sensors due to their low activation temperature [161,162].

### 6.2. Boronic/Boronate Ester Bond

**Figure 18:**
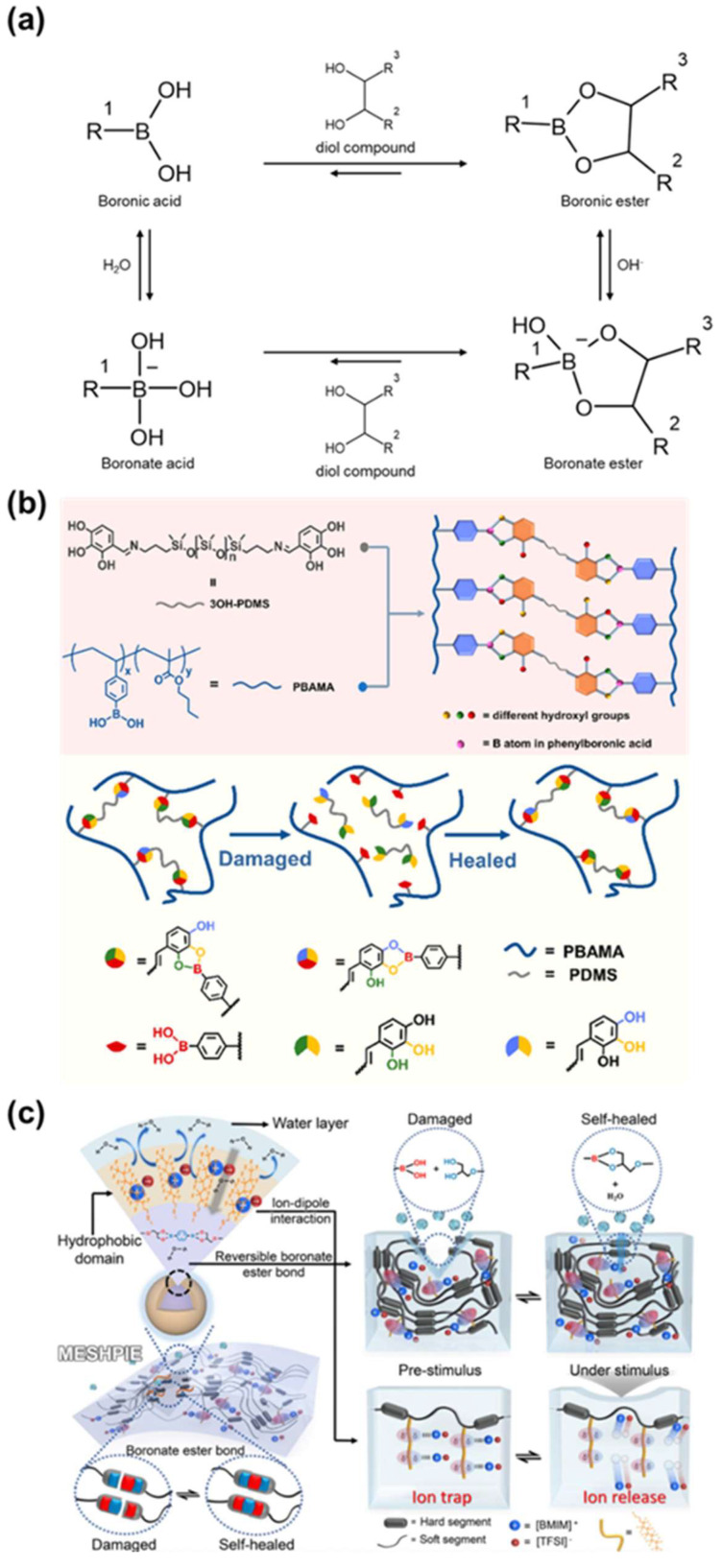
Boronic/boronate ester (**a**) Structure and mechanism of boronic/boronate ester bond. (**b**) Synthesis of phenolic compounds based on dynamic B–O bonding using pyrogallol with three hydroxyl groups. Reprinted/adapted with permission from Ref. [168]. 2021, American Chemical Society. (**c**) Application to an ultrafast self-healing piezo-ionic elastomer capable of functioning in both air and underwater environments. Reprinted/adapted with permission from Ref. [173]. 2024, Nature.

Boronic/boronate esterification is a reaction in which boronic acid or boronate reacts with diol to form boronic/boronate ester (Figure 18a) [163]. Characteristic B–O bonds are formed depending on the pH or composition of the medium. Boronic acid is a Lewis acid that competes with the boronate acid form depending on its Lewis acidity and pKa [164]. Additionally, depending on the interaction with the solvent, the formation of boronate ester is preferred in aqueous solutions, while that of boronic ester is preferred in organic solvents. Dynamic properties of B–O bond formation and release occur through processes such as transesterification, hydrolysis-dehydration, and metathesis [165]. The efficiency of bond formation and release is modulated by molecular components and substituents involved [166,167].

Zhao et al. designed a phenolic compound considering the effect of the number of hydroxyl groups on the dynamic B–O bond (Figure 18b) [168]. They investigated the boronic transesterification of pyrogallol having three hydroxyl groups with boronic acid. While catechol (with two hydroxyl groups) undergoes only intermolecular exchange, pyrogallol allows for intramolecular exchange, facilitating dynamic dissociation and reassociation. Based on this, a crosslinked copolymer was synthesized by forming a boronic ester bond through boronic acid in poly(4-vinylphenylboronic acid-butyl methacrylate) (PBAMA) and pyrogallol in PDMS. Their research expanded the versatility and demonstrated the importance of boronic esterification by fabricating effective self-healing polymer materials through rational design at the molecular scale.

Boronate ester is capable of self-healing at room temperature, which leads to use in various fields, such as adhesives, electronics, biology, and medicine [169,170,171,172]. Kong et al. reported an ultrafast underwater self-healing piezo-ionic elastomer by designing a polymer that exhibits self-healing behavior in both air and water environments (Figure 18c) [173]. The polymer was fabricated based on a boronate ester linkage and C-F functionalized PU matrix. Self-healing was achieved through metathesis bond exchange reactions in air and through hydrolysis/re-esterification in water. The hydrophobic C-F group facilitated dynamic binding behavior in water. The elastomer achieved an impressive self-healing efficiency of 89.6% in 22 min in air and 15 min in water. Additionally, the introduction of ionic liquids expanded its use as a pressure-induced tactile sensor. Overall, molecular design using boronic/boronate ester has broadened application fields for materials that can operate effectively in diverse environments.

## 7. Summary and Perspective

Dynamic covalent bonds are powerful tools for reversibly achieving exchange, dissociation, and conversion in response to external stimuli, thus contributing to the development of smart polymers. Controlling the dynamic properties of polymers through temperature without catalysts is easy, cost-effective, and environmentally friendly. To summarize these advancements, Table 1 is presented that includes recent promising temperature-controlled dynamic polymers, as well as those introduced in this paper.

The dynamic hindered-urea bond allows for the control of substituent volume. It also effectively releases and forms bonds even at low temperatures. Disulfide bonds leverage the unique bond energy and characteristics of group 16 elements. In contrast, imine bonds featuring a double bond between carbon and nitrogen operate at relatively high temperatures. The Diels-Alder reaction is notable for producing cyclic molecules. These dynamic covalent bonds are applied in various fields, including adhesives, sensors, electronic devices, biology, and medicine.

A detailed perspective of the proposed dynamic covalent bonds reveals that the hindered-urea bond can operate at room temperature, facilitating bond formation and dissociation without harsh external stimuli. This attribute promotes its application under mild conditions, necessitating further research into its functionalities and potential uses in both industry and academia. The disulfide bond, a type of structural bond found in proteins, shows promise for applications in the bio-field of dynamic covalent chemistry when implemented in bio-derived polymer backbones. Conversely, selenides, although belonging to the same group as sulfur, exhibit significantly lower stability, requiring additional investigation. Imine bonds, while easily implemented, are unstable and readily dissociate under harsh chemical conditions. Thus, further research into their stability is still needed. Dynamic covalent bonds based on the Diels-Alder reaction offer stable bonding and the ability to adjust the healable temperature through different substituents, enabling flexible application across various external conditions. Despite the ongoing research on dynamic covalent bonds, further studies are also necessary to enhance their responsiveness to external stimuli and expand their functionality. Achieving these goals requires approaches and analyses from a molecular engineering perspective. Controlling the dynamic library and examining it through kinetic and thermodynamic perspectives can lead to the desired properties of polymers and create effective synthetic routes. Moreover, the ability to be applied not only in mild temperature conditions but also in various environments is required. Careful consideration is required for the design and synthesis of polymers with dynamic covalent bonds to achieve this. Dynamic covalent bonds that respond to temperature stimuli are anticipated to advance the development of adaptive and self-healing materials, leveraging molecular engineering insights and diverse functionalizations.

## Figures and Tables

**Table 1:** Dynamic properties of recently reported and promising polymers utilizing hindered-urea, disulfide, imine, and Diels-Alder bonds.

Dynamic Covalent Bond	Polymer Matrix	Stimulus	Properties	Application	Ref.
Hindered-urea Bond	PtB-HDI ^1^ and PtB-IPDI ^2^	5 h at 37 °C in the presence of water (self-healing)	Water-adaptability, self-healable	Self-healable tube for flowing water	[48]
	HUBM-co-PPGA ^3^	1 h at 80 °C (homolytic bond exchange) ~4 h at 120 °C (heterolytic bond exchange)	Shape memory, reprocessable, reprogrammable, thermally switchable	3D printing ink	[52]
	HUG-EP ^4^	4 h at 80 °C (crosslinked) 20 min at 160 °C (self-healing)	Shape memory, reprocessable, biodegradable recycling	Electronic packaging	[174]
	WPUU ^5^	2 h at 80 °C	Anti-corrosion, self-healable	Protective coating layer	[175]
	PG ^6^	30 min at ambient temperature, followed by 3 h UV light (36 w, 365 nm)	Mechanically robust, self-healable, weldability, anti-corrosion	Humidity sensor	[176]
	PUT ^7^	2 h at 50 °C followed by UV curing	Reprocessable, self-healable, solubility, thermal stability	UV-shielding	[177]
Disulfide Bond	PDSN ^8^	Heat at 80 °C, followed by UV light	Shape memory, self-healable, adhesive force	-	[87]
	Epoxy resin from BGPDS ^9^	30–60 min with DBU ^10^ at 100 °C or photo-irradiation (245 nm, 2 mW/cm^2^)	Rigid, degradable	-	[88]
	EPO345 ^11^-CTAM ^12^	well crosslinked above 80 °C	Adaptable network, reshapable, shear strength adhesive	Adaptable thermoset adhesive	[94]
	PU ^13^ polymers with TEA ^14^	56 °C (disulfide exchange), 1 h at 130 °C (self-healing)	Low hysteresis, stretchable, self-healable, crystallinity	EMG monitoring	[95]
	Polyurethane	70 °C (chain expansion)	Energy dissipation, segmental mobility, rigid	Damping elastomer	[96]
	Epoxy resin	1 h at 60 °C	Self-healable, high-strength, reprocessable	-	[91]
	Epoxy resin reinforced with fiber	5 min at 200 °C under 100 bar	Reprocessable, reparability, recyclable	Thermosets	[178]
Imine Bond	Vanilin-based epoxy resin	40 min at 170 °C under 0.3 MPa (self-healing)	Thermal and solvent resistivity, self-healable, recyclable	Bio-based curing agent	[15]
	PBS ^15^ oligomer	240 h at 80 °C	Biodegradable, Self-healable	-	[114]
	SH-BDA-EP ^16^	25 h at 50 °C under the acidic condition	Bio-derived, fire-resistance, degradable	Thermosets	[115]
	DACNF ^17^-PI ^18^-LCD ^19^	15 min at 80 °C under 3 MPa	Self-healable, Recyclable, water barrier property, solvent resistance	Bio-degradable plastic	[116]
	FDP ^20^/AMDP ^21^	pH = 12.1 (association), pH = 7.0 (dissociation)	Self-assemble, pH-sensitive, guest molecule loading	Bolaform superamphiphile	[117]
	Polybutadiene network	10 min at 80 °C (Recycle, Self-healing)	triple-shape memory effect, plasticity, recyclable, self-healable	multi-shape memory polymer	[118]
	Betulin-based polyurethane	40 min at 80 °C (Self-repairing)	Self-healable, reprocessable, degradable	editable shape memory polymer	[179]
	Benzoxazine monomer	5 min at 100 °C (Reshaping), 30 min at 140 °C under 2 MPa (Reprocessing)	Shape memory, reprocessable, degradable	shape memory polymer	[180]
Diels-Alder Bond	Crosslinked Polyurethanes	3 h at 160 °C, followed by 20 h at 70 °C	Recyclable	-	[140]
	DOPO ^22^-FU ^23^-BMI ^24^	1 h at 150 °C, followed by 12 h at 95 °C.	Self-healable, flame retardancy	-	[146]
	DOPO-TMP ^25^-EP ^26^	30 min at 180 °C	Self-healable, flame retardancy, recyclable	-	[147]
	Urushiol-based coating	30 min at 125 °C	Self-healable, hydrophobicity, hardness	Self-healing coatings	[150]
	Photothermally activated nanofibers	NIR light for 15 min	Drug release controllable	Drug delivery	[153]
	Lignin-derived elastomers	20 min at 120 °C	Self-healable, thermal stability, shape memory	Hot-melt adhesives	[181]
	PEG crosslinked by DABBF ^27^	40 °C	Recyclable, plasticity, self-healable	-	[182]

^1^ Linear prepolymers crosslinked with 1,6-diisocyanatohexane; ^2^ linear prepolymers crosslinked with isophorone diisocyanate; ^3^ a hindered urea containing bismethacrylate and poly(propylene glycol); ^4^ epoxy samples containing hindered urea bonds; ^5^ waterborne polyurethane urea composite coatings; ^6^ poly(urethane-urea)-Glycidyl methacrylate; ^7^ the hindered-urea bond containing thermosets; ^8^ polydisulfide network; ^9^ bis(4-glycidyloxyphenyl)disulfide; ^10^ 1,8-Diazabicyclo [5.4.0]undec-7-ene; ^11^ poly[(phenyl glycidyl ether)-co-formaldehyde] (Mn ≈ 570 g/mol); ^12^ Cystamine; ^13^ polyurethane; ^14^ triethanolamine; ^15^ poly(butylene succinate); ^16^ diglycidyl ether of 1,4-butanediylbis (4hydroxy-3,5-dimethoxybenzylidene)imine); ^17^ dialdehyde cellulose nanofibrils; ^18^ polyimide; ^19^ long-chain diamine monomers; ^20^ 1-(10-(4-Formylphenoxy)decyl)pyridinium; ^21^ 1-(10-(4-(Ammoniomethyl)phenoxy)decyl)- pyridinium; ^22^ 9,10-dihydro-9-oxa-10-phosphaphenanthrene-10-oxide; ^23^ Furfural; ^24^ 1,1′-(methylenedi-4,1-phenylene)bismaleimide; ^25^ tri(4-maleimidophenol) phosphate; ^26^ epoxy resin; ^27^ diarylbibenzofuranone.

## Data Availability

Data are contained within the article.
